# Profiling of mRNA of interstitial fibrosis and tubular atrophy with subclinical inflammation in recipients after kidney transplantation

**DOI:** 10.18632/aging.102115

**Published:** 2019-07-25

**Authors:** Qiang Fu, Minxue Liao, Cheng Feng, Jichao Tang, Rui Liao, Liang Wei, Hongji Yang, James F. Markmann, Kai Chen, Shaoping Deng

**Affiliations:** 1 Organ Transplantation Center, Sichuan Provincial People's Hospital and School of Medicine of University of Electronic Science and Technology of China, Chengdu 610072, Sichuan, China; 2 Transplant Center, Massachusetts General Hospital, Harvard Medical School, Boston, MA 02148, USA; 3 Organ Transplantation Translational Medicine Key Laboratory of Sichuan Province, Chengdu 610072, Sichuan, China; 4 North Sichuan Medical College, Nanchong 637100, Sichuan, China; 5 Southwest Medical University, Luzhou 646000, Sichuan, China

**Keywords:** kidney transplantation, interstitial fibrosis and tubular atrophy with subclinical inflammation (IFTA-I), interstitial fibrosis and tubular atrophy (IFTA), IRF8

## Abstract

Interstitial fibrosis and tubular atrophy (IFTA) with inflammation (IFTA-I) is strongly correlated with kidney allograft failure. Diagnosis of IFTA-I accurately and early is critical to prevent graft failure and improve graft survival. In the current study, through analyzing the renal allograft biopsy in patients with stable function after kidney transplantation (STA), IFTA and IFTA-I group with semi-supervised principal components methods, we found that CD2, IL7R, CCL5 based signature could not only distinguish STA and IFTA-I well, but predict IFTA-I with a high degree of accuracy with an area under the curve (AUC) of 0.91 (*P* = 0.00023). Additionally, IRF8 demonstrated significant differences among STA, IFTA and IFTA-I groups, suggesting that IRF8 had the capacity to discriminate the different classifications of graft biopsies well. Also, with Kaplan-Meier and log-rank methods, we found that IRF8 could serve as the prognostic marker for renal graft failure in those biopsies without rejection (AUC = 0.75) and the recipients expressing high had a higher risk for renal graft loss (*P* < 0.0001). This research may provide new targets for therapeutic prevention and intervention for post-transplantation IFTA with or with inflammation.

## INTRODUCTION

After the 1^st^ year post-transplant, approximately 3% of all kidney allograft recipients manifest allograft dysfunction [1]. This contributes significantly to higher morbidity and mortality [2]. Only a small fraction of those recipients are able to receive renal allograft re-transplantation because of the shortage of donor organs [3]. Though immunosuppression regimens have significantly decreased acute rejection, the 10-year graft survival remains unchanged [4, 5]. Therefore, novel approaches to improve long-term allograft survival are needed.

Interstitial fibrosis and tubular atrophy (IFTA) are common histological abnormalities and are present in about 25% of 1-year surveillance biopsies after kidney transplantation. It has been reported that IFTA with inflammation (IFTA-I) is strongly correlated with kidney allograft failure [6]. Since the influence of IFTA-I on graft outcomes was first suggested in 2009, numerous researchers and clinicians have reported the detrimental impact of IFTA-I [5–7]. Also, it has been reported that IFTA-I is associated with under-immunosuppression and typically preceded by chronic T cell-mediated rejection (TCMR) [8, 9]. While histological assessment of ITFA-I according to the Banff classification at that time suffered from poor inter-observer reproducibility and lacked the sensitivity for the early stage IFTA with or without inflammation [7], thereafter, at the Banff Conference in 2017, the clinical implications of IFTA-I and its relationship to TCMR came into focus. The Banff classification of renal allograft pathology was revised to include moderate IFTA-I plus moderate or severe tubulitis as diagnostic of chronic active TCMR [10]. Currently, diagnosis of IFTA mainly depends primarily on renal allograft pathology and some other techniques such as Fourier-Transform Infrared Imaging Technique [7], However, the former showed high variation among different institutions, which may cause different diagnostic results [11]. The high variation of biopsy results may confuse the treatment strategies, which could mislead to the intervention of allograft rejection process.

In this study, we analyzed the genome-wide RNA-Seq profiles to assess whether there was key marker(s) in the renal allograft biopsy with IFTA-I that could aid in the diagnosis and prognosis of IFTA-I qualitatively and quantitatively. Using Protein-protein interaction (PPI) and ROC analysis, we found that a 3-gene signature (CD2, IL7R, CCL5) had a high degree of accuracy, in diagnosing IFTA-I, the area under the curve (AUC) being 0.91. Additionally, IRF8 could be used as the prognostic marker (AUC 0.75, *P* < 0.00001). It is conceivable that these biomarkers could be targeted to prevent disease progression in the future.

## RESULTS

### Identification and analysis of differentially expressed genes (DEGs) 

**Figure 1:**
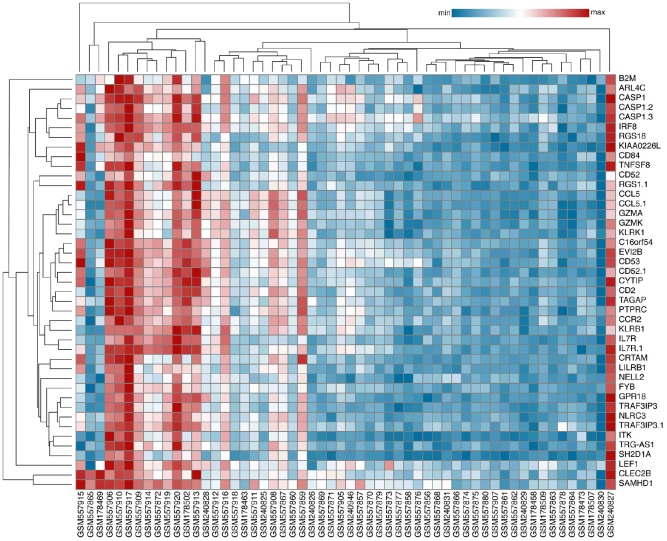
**Identification of Key DEGs.** A total of 43 DEGs were identified with an SPC method and random forest.

After inclusion and exclusion, GSE22459, GSE9489, GSE7392, and GSE9492 were selected and the clinical information was listed in the Supplementary Tables 1–5 [12–14]. A total of 56 samples, including 40 samples with stable function after kidney transplantation (STA) and 16 IFTA-I ones were integrated and normalized and a total of 249 transcripts with log2 fold change (log2 |FC|) >1, FDR adjusted *P* < 0.001) were identified finally (Supplementary Figure 1). All those transcripts were up-regulated. Then 43 DEGs were identified with a semi-supervised principal components (SPC) method and random forest with a threshold of Mean Decrease Gini >0.1 (Figure 1), the biological and the genetic and biological function table of those 43 genes was provided in the Supplementary Table 6.

### Validation of DEGs

**Figure 2:**
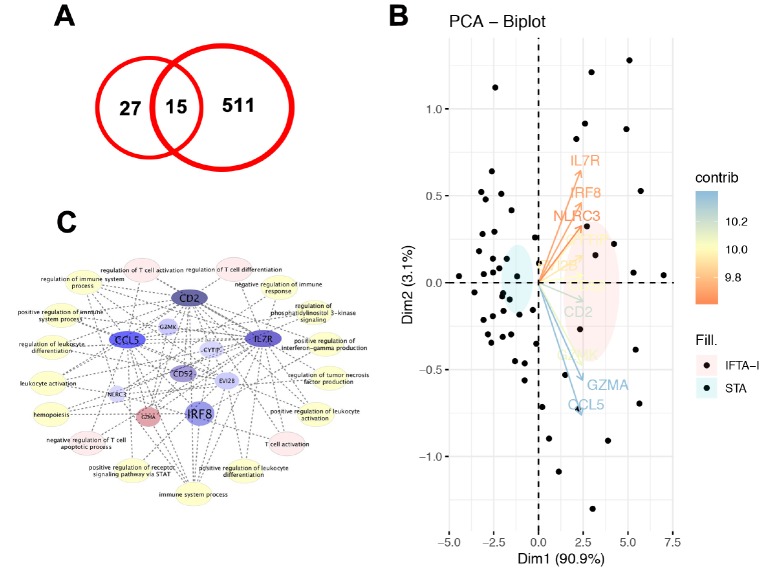
**Validation of DEGs.** (**A**) A total of 15 common DEGs were up-expressed or down-expressed simultaneously in both databases; (**B**) PCA analysis found that the contribution degrees of 10 DEGs, including EVI2B, CYTIP, GZMK, CD2, IRF8, IL7R, CD52, NLRC3, GZMA, and CCL5, were all > 0.9; (**C**) In those 10 genes, MCODE scores of CD2, IL7R, and CCL5 were all > 3 and the numbers of their nodes were all > 4.

With a similar method, a total of 526 DEGs were identified in GSE 76882, and 15 common DEGs which were up-expressed or down-expressed simultaneously in both databases (Figure 2A). Principal component analysis (PCA) found that 10 transcripts, including EVI2B, CYTIP, GZMK, CD2, IRF8, IL7R, CD52, NLRC3, GZMA and CCL5, the contribution degrees were all > 0.9 (Figure 2B).

### Identification of key genes

**Figure 3:**
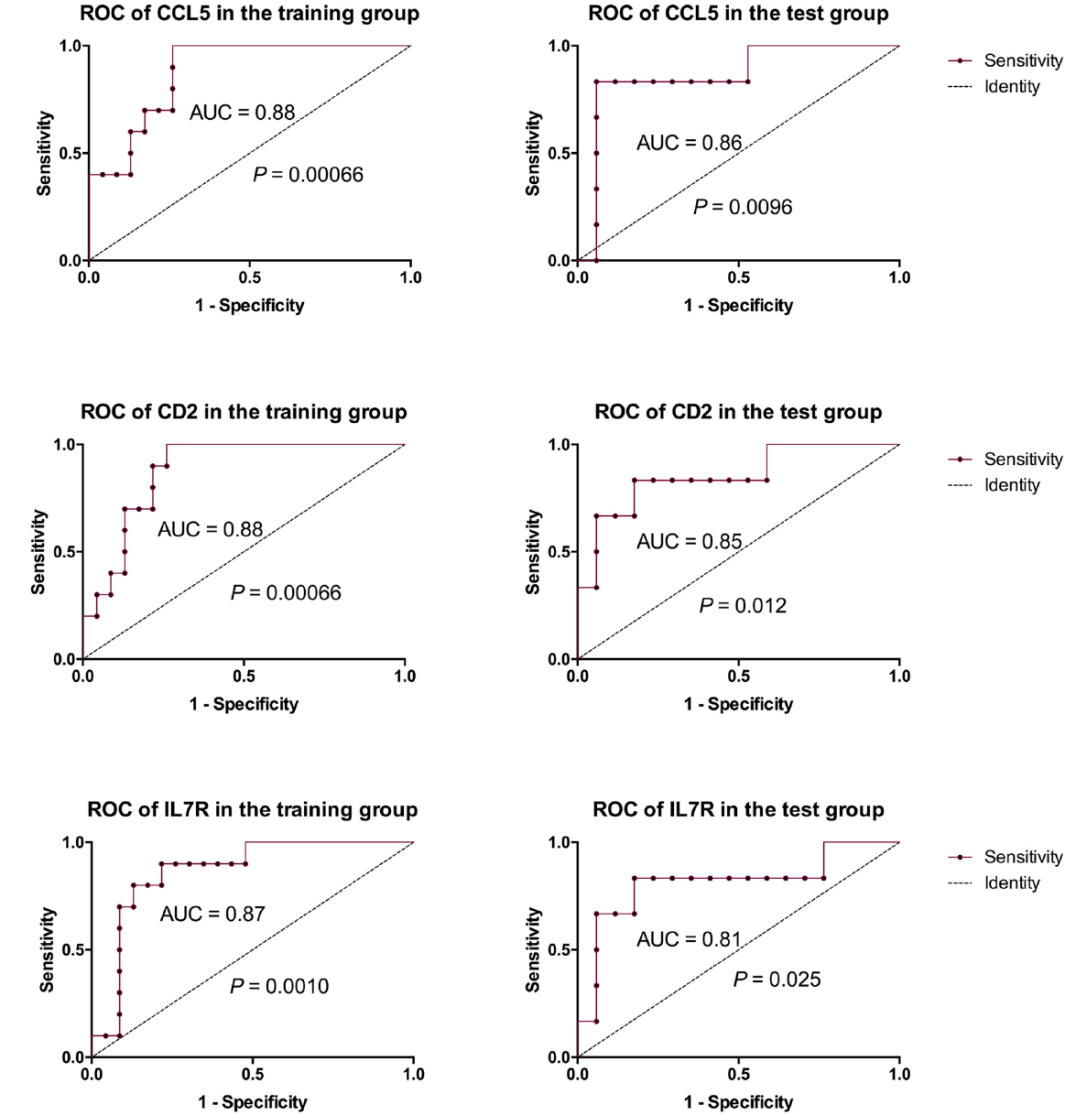
**ROC analysis of 3 key genes.** ROC analysis showed that AUC of CD2, IL7R and CCL5 in both training and test groups were all > 0.8.

In the PPI analysis, we found that plug-in Molecular Complex Detection (MCODE) scores of CD2, IL7R, and CCL5 were all > 3 and the numbers of their nodes were all > 4. Also, those 3 genes were enriched in the most abundant signals, including T cell activation, regulation of T cell activation and differentiation and negative regulation of T cell apoptotic process and so on (Figure 2C). ROC analysis showed that AUC of CD2, IL7R, and CCL5 in both training and test groups were all > 0.8 (Figure 3).

### Prognostic model establishment based on a 3-gene signature

**Figure 4:**
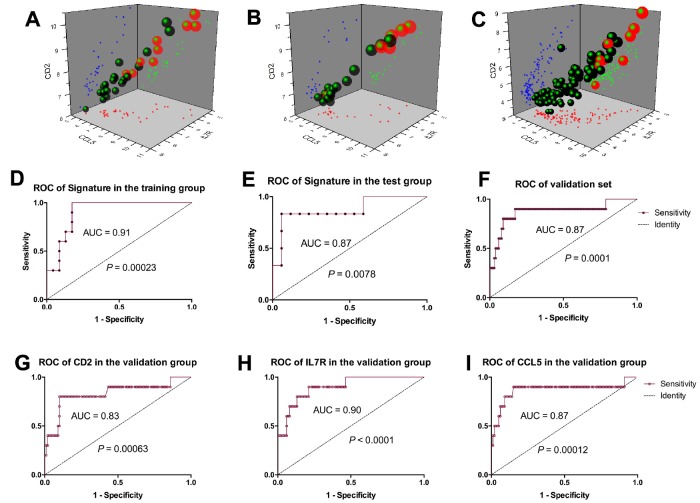
**Prognostic model establishment based on the 3-gene signature.** Scatter diagram of the training group. (**A**), test group (**B**), and validation group (**C**) showed the subjects with higher prognostic scores showed a tendency towards the expression of high-risk genes. ROC curves of the training group, test group and validation group were respectively 0.91(**D**), 0.87 (**E**) and 0.87 (**F**); ROC curves of CD2, IL7R and CCL5 were respectively 0.83 (**G**), 0.9 (**H**) and 0.87 (**I**).

Thereafter, we developed a prognostic model for IFTA-I diagnosis. According to the ROC curve, the optimum cutoff point was calculated and used for classifying the samples into high-risk and low-risk groups. Scatter diagram indicated that the subjects with higher prognostic scores showed a tendency towards the expression of high-risk transcripts (Figure 4A, 4B). ROC curves of the training group and test group were respectively 0.91 and 0.87 (Figure 4D, 4E).

### Three-gene signature model validation

Scatter diagram of the validation group showed the subjects with higher prognostic scores showed a tendency towards expression of high-risk transcripts (Figure 4C). ROC curve of the validation group revealed that the AUC of 3-gene signature was 0.87 (*P* = 0.0001, Figure 4F), CD2, IL7R and CCL5 were 0.83 (*P* = 0.00063), 0.90 (*P* < 0.0001) and 0.87 (*P* = 0.00012) respectively (Figure 4G–4I).

### IRF8 as the key genes for Identification of IFTA-I between IFTA

**Figure 5:**
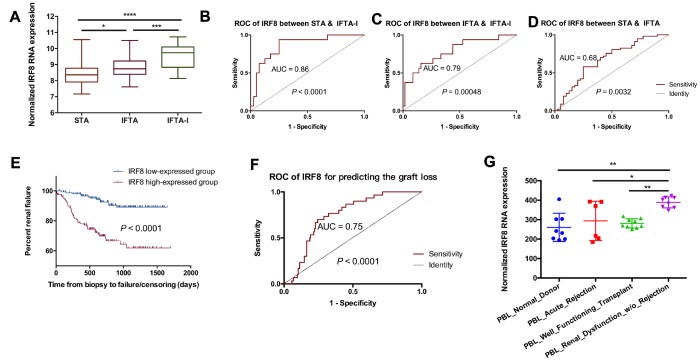
**IRF8 as the identification of IFTA-I between IFTA**. (**A**) IRF8 expression had significant differences among the STA, IFTA and IFTA-I groups; ROC curve showed that IRF8 could be used for predicting IFTA-I from STA (**B**) and IFTA (**C**) and STA from IFTA (**D**); (**E**) All biopsies were divided into IRF8 high-expressed and low-expressed group and it indicated that recipients with high IRF8 expression were easier to develop into renal graft dysfunction and failure than that in the IRF8 low-expressed group (*P* < 0.00001); (**F**) AUC of IRF8 was 0.75 in the biopsies without rejection; (**G**) IRF8 expression was higher-expressed in the peripheral blood lymphocyte (PBL) in renal dysfunction w/o rejection than those with normal kidney function or with acute rejection in post-transplantation recipients. *P < 0.05, **P < 0.01, ***P < 0.001, ****P < 0.0001.

Though the 3-gene set could have a significant expression between the IFTA-I and STA groups while not between the IFTA and STA group. It means that the 3-gene signature above may not differentiate IFTA from STA well. To further identify the difference among STA, IFTA-I, and IFTA, DEGs among STA, IFTA and IFTA-I groups were selected as the key genes. There were 7 DEGs (EVI2B, GZMK, IRF8, IL7R, CCL5, CD52, and GZMA) showed significant differences among those three groups (*P* < 0.05, Figure 5A and Supplementary Figure 2). ROC curves of IRF8 (Figure 5B–5D), CCL5, CD52, EVI2B, GZMA, GZMK, and IL7R (Supplementary Figure 3) showed high AUC for distinguishing IFTA from IFTA-I. To confirm if those genes could be used as prognostic markers for renal graft failure, all biopsies were divided into high-expressed and low-expressed groups with the optimal cutoff point of the prognostic score obtained in ROC curve analysis (Figure 5E and Supplementary Figure 4). The result indicated that recipients with high IRF8 expression were easier to develop into renal graft dysfunction and failure than that in the IRF8 low-expressed group (*P* < 0.00001) and AUC was 0.75 in the biopsies without rejection (Figure 5F). In addition, we found that IRF8 expression was higher-expressed in the peripheral blood lymphocyte (PBL) in renal dysfunction w/o rejection (Figure 5G) than those with normal kidney function or with acute rejection in post-transplantation recipients. It meant that IRF8 may promote graft loss, and it may be potentially used for identification of graft loss through peripheral blood in the future.

### DISCUSSION

Comparing with IFTA, IFTA-I has shown a stronger correlation with kidney allograft dysfunction and renal graft failure. Diagnosing IFTA-I accurately is therefore critical to prevent graft failure and improve graft survival. In the current study, through analysis of the renal allograft biopsy in the STA, IFTA, and IFTA-I groups, we found that CD2-IL7R-CCL5 based signature not only differentiated separate STA and IFTA-I but also predicted IFTA-I with a high degree of accuracy (AUC = 0.91). These results suggest that measuring the expression of CD2, IL7R, and CCL5 expression in renal allograft biopsies, we can learn about the risk of the IFTA-I occurrence. Additionally, IRF8 expression correlated with significant differences among STA, IFTA, and IFTA-I, suggesting that IRF8 could be used to classify graft biopsies into different risk groups. Also, IRF8 may serve as a valuable prognostic marker for renal graft failure in those biopsies without rejection and as the therapy target (AUC = 0.75).

CD2 is considered as a trigger of T cell-mediated immunoreaction [15]. It has been reported that allo-responsive CD8^+^CD2^hi^CD28^−^ T cells which are the most functionally active T cell subset, comparing with CD8^+^CD2^low^CD28^+^ and CD8^+^CD2^hi^CD28^+^ T cells, can express higher poly-functional cytokines and cytotoxic effector molecules such as IFN-c, TNF, IL-2 and CD107a and so on [16]. Selectively targeting CD2 in human allo-responsive CD8^+^CD2^hi^CD28^−^ T cells can reduce costimulation blockade-resistant cells and prolong renal allograft survival [16]. Tatsuo Kawai’s group found that targeting CD2 therapy could successfully delay the expansion of CD2^hi^ cells including CD8^+^CD95^+^ effector memory T (T_EM)_ cells while sparing CD8^+^CD95^+^CD28^-^ naive T and NK cells and promote the donor hematopoietic chimerism development and prolong immunosuppression-free renal graft survival in nonhuman primates [17]. This finding was consistent with previous studies [18]. We found that CD2 was highly expressed in renal graft biopsies with IFTA-I comparing with STA and IFTA renal graft biopsies. This suggests that CD2 may play an important role in IFTA-I development. Therapy targeting CD2 may prove useful in manifesting recipient IFTA-I post-transplantation. IL7R, also known as CD127 or IL7 alpha, was first reported that in 2006 had an opposite expression and capacity in Treg suppression comparing with FoxP3 [19]. Though monitoring the balance of CD4^+^CD25^hi^IL-7R^hi^Foxp3^-^ and CD4^+^CD25^hi^FoxP3^+^IL-7R^low^ T cells in renal transplant recipients, Manuel Pascual group found when chronic humoral rejection occurred, a strikingly higher proportion of CD4^+^CD25^hi^IL-7R^hi^Foxp3^-^ T cells were present [20]. It was also reported that CD127^low^ Expression in CD4^+^CD25^high^ T Cells could be used as the immune biomarker of renal function in transplant patients [21]. While the results showed that the recipients with a higher frequency of CD127^low^ cells owed a high deterioration of renal function [21]. Giuseppe Pantaleo group reported that CD4^+^CD25^hi^IL-7R^hi^CD45^RO^ cells infiltrating the allograft of patients and could expand significantly in patients with a documented diagnosis of chronic humoral rejection [22]. In this study, IL7R were highly expressed in IFTA-I recipients that those with IFAT or STA recipients, which was in accordance with the study mentioned above. CCL5, also called RANTES, was reported in early 1994 that could be highly expressed in cell-mediated transplant rejection in renal recipients and had the potential as the prime target for immunomodulation in transplant rejection [23]. It was also reported that CCL5 could help to identify grafts with early acute antibody-mediated rejection [24]. Daniel Maluf group found that CCL5 was overexpressed in IFTA comparing with normal allografts and kidneys [25]. In the current study, we found that there was a difference (*P* < 0.05) among the STA, IFTA, and IFTA-I groups. CCL5 could distinguish IFTA-I from IFTA and STA biopsies well. However, CCL5 could not be used as the prognostic marker for graft loss, which was in accordance with previous reports [26].

As a contrast, IRF8 could effectively separate IFTA-I, IFTA and STA as prognostic markers for graft loss. However, there was no study on IRF8 function in kidney transplantation. As a transcription factor expressed throughout B cell differentiation, IRF8 could contribute to molecular crosstalk among germinal center B cells, T follicular helper cells, and follicular dendritic cells [27]. Also, IRF8^-/-^macrophages were impaired in the activation of Toll-like receptor signaling and the production of proinflammatory cytokines such as IL-1, IL-6, IL-12 [28]. It means that IRF8 may have the potential to be used as the biomarker for IFTA-I.

Though we confirmed that the significant prognosis role of IRF8 in the renal graft recipients IFTA with or without inflammation, our study was limited because the number of kidney transplantation recipients was small, the study was retrospective, and the underlying pathological mechanisms of the genes identified remain speculative. Ultimately, the prognostic potential of the IRF8 should be further tested prospectively in a larger analysis of kidney transplant recipients before wider application.

In conclusion, we confirmed IRF8 expression as an accurate diagnostic and prognostic biomarker for the renal recipients with IFTA, with or without inflammation post-transplant. We are currently working to confirm the diagnostic effect of IRF8 in the prospective study and then elucidate the underlying mechanisms by which IRF8 may function in this setting. We hypothesize as actions through regulation of T follicular helper and T follicular regulatory cell balance. This research may provide a new therapeutic target for the prevention of post-transplantation IFTA with or with inflammation.

## MATERIALS AND METHODS

### The microarray datasets

Microarray data Gene expression profiles of allograft protocol biopsies were obtained from the GEO database (https://www.ncbi.nlm.nih.gov/geo/). Because the Annotation files may be different when GPL platform varies, only the study from the same platform could be combined to study. Also, The more programs of the GPL platform, the more mature of the technology. The GPL with maximum numbers, that is Agilent GPL570 platform [HG-U133_Plus_2], was selected for the study. Based on GPL570, the samples which were diagnosed as Stable function after kidney transplantation (STA), chronic Interstitial fibrosis and tubular atrophy (IFTA) or IFTA with inflammation (IFTA-I) and more than 6 months post-transplant, based on pathology, were included. Those samples without STA, chronic IFTA or IFTA-I diagnosis and those with IFTA or IFTA based on pathology but less than 6 months post-transplant, were excluded. Finally, GSE22459, GSE7392, GSE9489, and GSE9492 were included, which contained 56 samples, including 40 allograft renal biopsies classified with STA and 16 biopsies with IFTA-I. The clinical information was listed in the Supplementary Tables 1–5 [12–14]. The outcomes were validated using GSE76882, based on GPL13158 [HT_HG-U133_Plus_PM].

### Identification of dysregulated transcripts in IFTA-I

All gene expression data were integrated and normalized by the gcRMA algorithm [29]. Then surrogate Variable Analysis and Empirical Bayes method were used to reduce batch effects and detect differentially expressed genes (DEGs) [30, 31]. Genes with log2 fold change (log2 FC) < −1 or log2 FC > 1 (FDR adjusted *P* < 0.001) were considered as DEGs and analyzed with principal component analysis (PCA) with R version 3.4.3.

### Identification of key DEGs in IFTA-I

To identify key DEGs related to prognosis, the 56 samples were randomly divided into a training and testing set at 6:4. A semi-supervised principal components (SPC) method and the random forest were used to select significant genes (Hazard Ratios > 1 or < 1 with *P* < 0.05 was used as the cutoff) [32, 33]. Then the key gene expression was validated with GSE76882 [5] and the genes contribution degree > 0.9 were selected. Then the common DEGs which were up-expressed or down-expressed simultaneously were defined as the key genes.

### PPI network and module analysis

PPI was analyzed according to the previous report [34]. Briefly, DEGs were mapped to STRING (http://string-db.org) database and those validated interactions with a combined score < 0.4 were selected and PPI networks were constructed and screened using the plug-in Molecular Complex Detection (MCODE, Cytoscape software). The key genes whose MCODE score > 3 and the number of nodes > 4 were included.

### Prognostic model by 3 genes based signature

The prognostic score was calculated in the training group as follows: Prognostic score = (3.833 × CCL5) + (6.133 × IL7R) + (4.14 × CD2). Therefore, the 3-gene signature was developed using the linear signature prognostic model. The optimal cutoff point of the prognostic score was obtained in ROC curve analysis for predicting the accuracy of the signature in the test group and in GSE76882 database [5].

### Identification of IFTA-I between IFTA and survival analysis

To distinguish IFTA-I from IFTA, DEGs among STA, IFTA, and IFTA-I groups were selected for further analysis. The optimal cutoff point of the prognostic score was obtained in ROC curve analysis and used for dividing the biopsies into high-expressed and low-expressed groups. Then Kaplan-Meier and log-rank methods were used for predicting renal graft failure in GSE21374 database [35]. To further confirm the expression of the key genes, we validated the key gene expression in GSE1563 database [36].

## Supplementary Material

Supplementary Figures

Supplementary Tables

Supplementary Table 6
