# Supplementary material for: Profiling of mRNA of interstitial fibrosis and tubular atrophy with subclinical inflammation in recipients after kidney transplantation
Source: Aging (Albany NY). 2019 Jul 25;11(14):5215–31. doi: 10.18632/aging.102115 (PMC6682514; doi:10.18632/aging.102115)
Supplement: Supplementary Figures [file aging-11-102115-s003.pdf]

SUPPLEMENTARY MATERIALS

Supplementary Figures

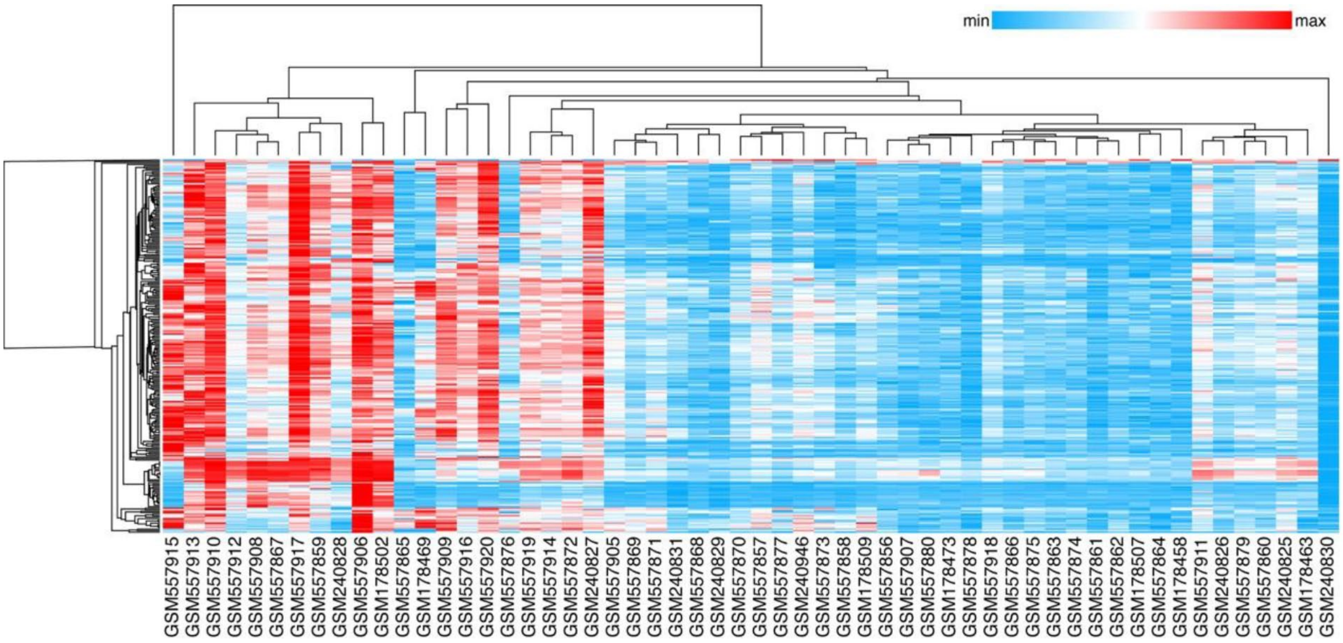

Supplementary Figure 1. Identification and analysis of DEGs. After the inclusion and exclusion, a total of 249 genes with log2 fold change (log2 |FC|) >1, FDR adjusted *P* < 0.001) were identified finally. All those genes were up-regulated.

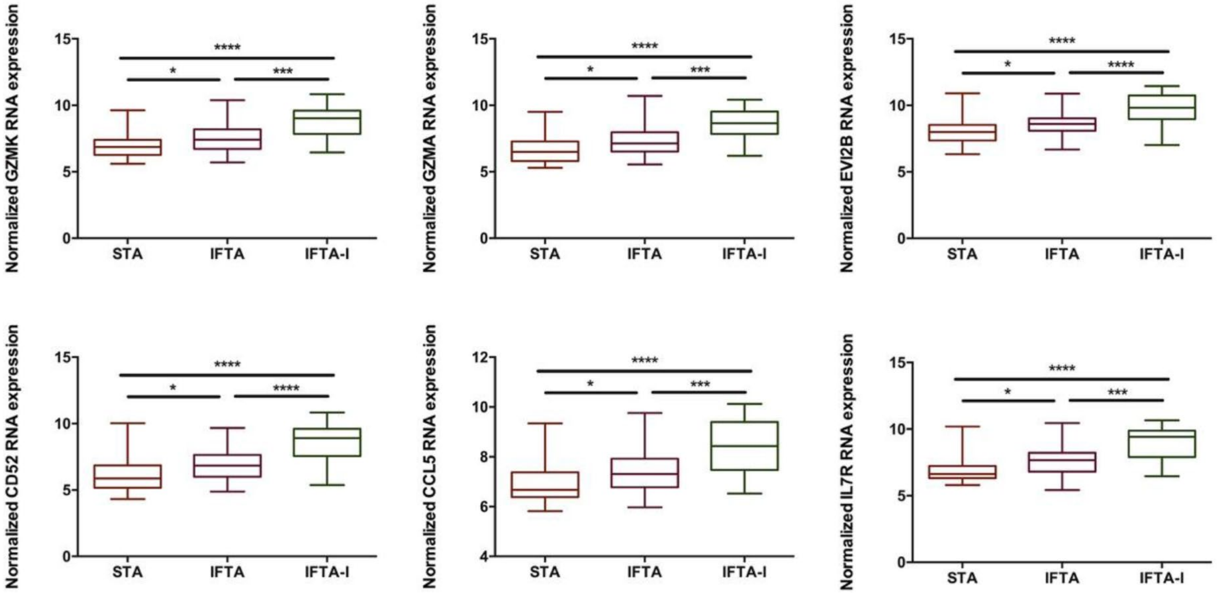

Supplementary Figure 2. RNA expressions of the key genes. The gene expressions of GZMK, GZMA, EVI2B, CD52, CCL5, and IL7R had significant differences among the STA, IFTA, and IFTA-I groups. **P* < 0.05, ****P* < 0.001, *****P* < 0.0001.

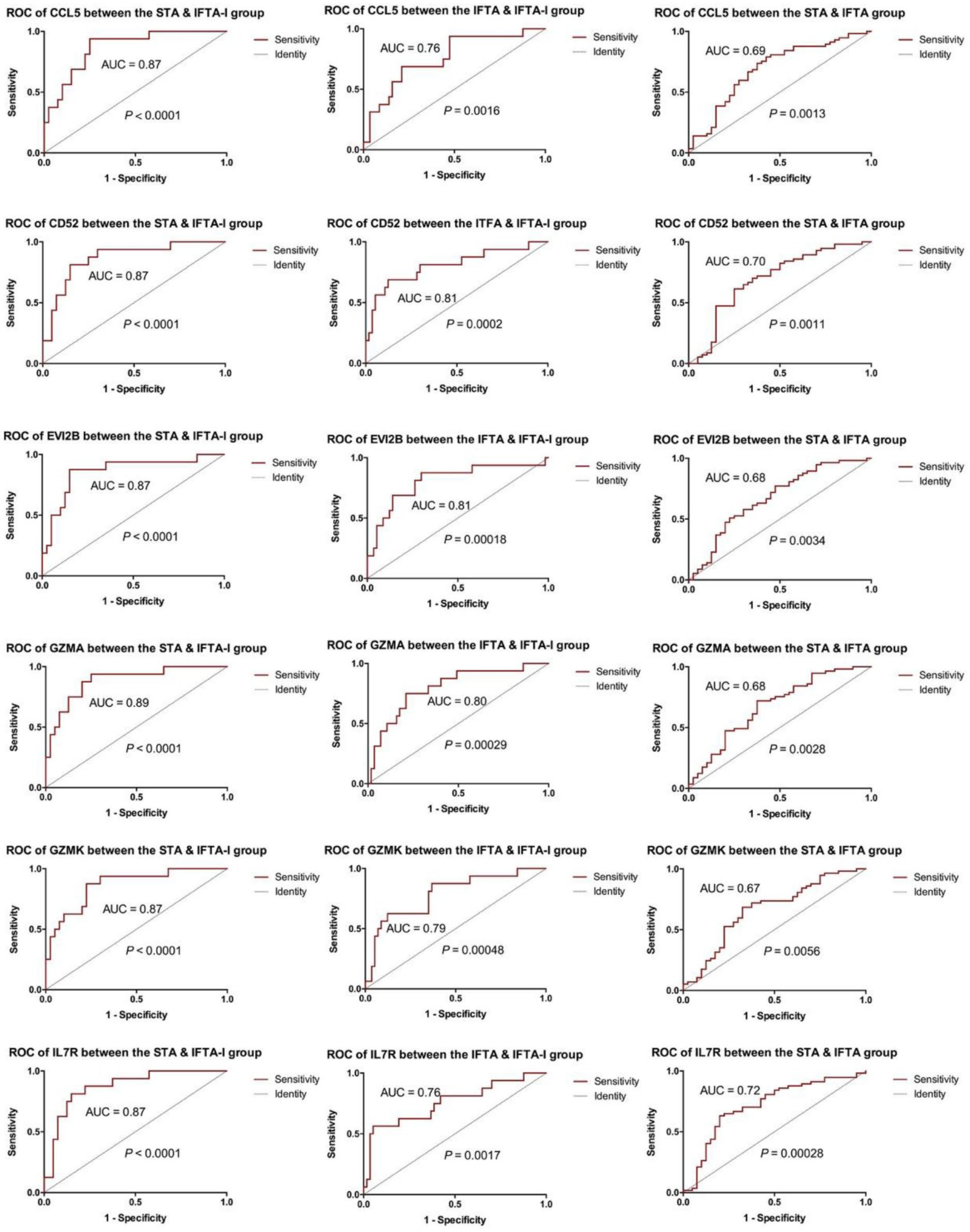

Supplementary Figure 3. ROC curve of the key genes. ROC curves showed that CCL5, CD52, EVI2B, GZMA, GZMK, and IL7R could be also used for differentiate IFTA-I from IFTA and STA respectively.

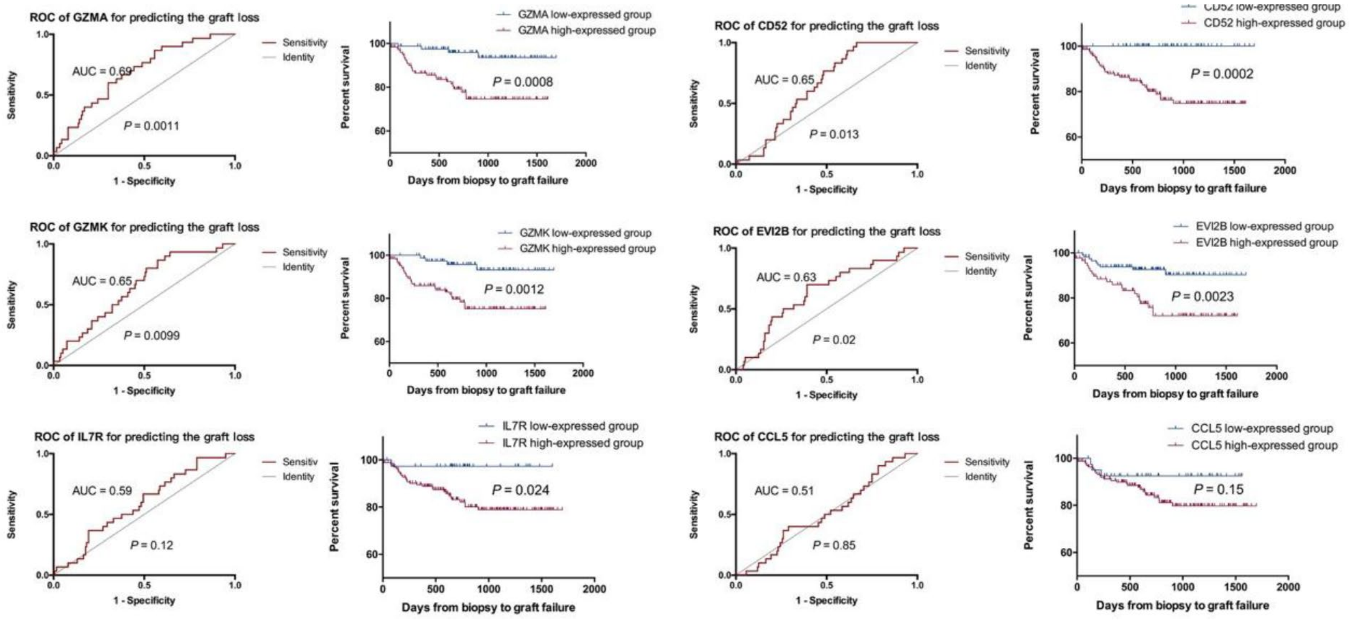

Supplementary Figure 4. ROC and graft loss prediction analysis. ROC and survival analysis of GZMA, CD52, GZMK, EVI2B, IL7R, and CCL5.
