# Supplementary material for: Profiling of mRNA of interstitial fibrosis and tubular atrophy with subclinical inflammation in recipients after kidney transplantation
Source: Aging (Albany NY). 2019 Jul 25;11(14):5215–31. doi: 10.18632/aging.102115 (PMC6682514; doi:10.18632/aging.102115)
Supplement: Supplementary Tables [file aging-11-102115-s002.pdf]

Supplementary Tables

Supplementary Table 1. Demographic and clinical characteristics of the biopsies for clinical indications according to histologic analysis in GSE22459.

Characteristic	Normal	IFTA	IFTA-I	P
***n* %**	57	30	13	NS
**T12 surveillance biopsy scores, original report (mean ± SD)**				
**g**	0.0 ± 0.0	0.0 ± 0.0	0.3 ± 0.6	NS, 0.002, 0.002
**i**	0.0 ± 0.0	0.0 ± 0.0	1.4 ± 0.6	NS, 0.0001, 0.0001
**t**	0.0 ± 0.0	0.0 ± 0.0	1.3 ± 1.0	NS, 0.0001, 0.0001
**ci**	0.0 ± 0.0	1.2 ± 0.5	1.2 ± 0.5	0.0001, 0.0001, 0.9
**ct**	0.0 ± 0.0	1.2 ± 0.5	1.2 ± 0.7	0.0001, 0.0001, 0.7
**cv**	0.3 ± 0.5	0.8 ± 0.7	0.5 ± 0.8	0.0001, 0.4, 0.1
**ah**	0.1 ± 0.3	0.3 ± 0.5	0.5 ± 0.8	0.001, 0.1, 0.9
**T12 surveillance biopsy scores, blinded reread (mean ± SD [% with score >1])**				
**total i**	ND	0.07 ± 0.26 (6%)	1.81 ± 0.93 (100%)	–, –, 0.0001
**Age at Tx (years, mean ± SD)**				
**Recipient**	52 ± 12	51 ± 15	47 ± 17	0.5
**Donor**	40 ± 11	44 ± 12	46 ± 14	0.1
**Female gender (%)**				
**Recipient**	45	36	55	0.3
**Donor**	55	47	55	0.7
**GFRu/2 (C_{ioth} ml/min; mean ± SD)**	59 ± 11	60 ± 12	55 ± 11	0.2

Supplementary Table 2. Demographic and clinical characteristics of the biopsies for clinical indications according to histologic analysis in GSE9489.

Characteristic	STA	IFTA	P
No. biopsies	7	22	NS
No. patients	7	22	NS
Recipient age (years)	43.1 ± 8.7	46.9 ± 12.2	NS
Recipient gender (*n*, % male)	6 (86%)	15 (68.2%)	NS
Donor age (years)	45.2 ± 15.4	43.4 ± 17.1	NS
No. HLA mismatches	1.8 ± 1.5	2.9 ± 1.3	NS
No. historic AR episodes (% patients with ‡1)	14.3	36.4	NS
Time of biopsy (months post-Tx)	25.1 ± 51.4	83.2 ± 64.8	0.042
No. patients with CNI toxicity (histology)	1	3	NS
No. patients on a CNI-free regimen	0	2	NS
Serum creatinine (lmol/l)	160.0 ± 44.4	281.6 ± 204.6	NS
GFR MDRD-calculated (ml/min/1.73 m^2)	43.0 ± 15.5	27.6 ± 13.5	0.017

Supplementary Table 3. Demographic and clinical characteristics of the biopsies for clinical indications according to histologic analysis in GSE76882.

Characteristics	IFTA	IFTA-I	Tx	*P*
Donors				
Age (mean ± SE)	40 ± 2	41 ± 5	41 ± 2	0.8
Female	23 (58%)	5 (50%)	37 (47%)	0.61
Black	5 (13%)	0	4 (5%)	0.39
Recipients				
Total number of patients	40	10	82	N/A
Total number of biopsies	42	10	99	N/A
IFTA Grade (Banff) 1/2/3	18/16/6	4/4/0	N/A	0.67
Protocol biopsy	13/40 (33%)	3/10 (30%)	72/82 (88%)	<0.0001
Age (mean ± SE)	44 ± 2	49 ± 5	50 ± 2	0.02
Female	19 (48%)	3 (30%)	28 (34%)	0.55
African American	4 (10%)	1 (10%)	6 (7%)	0.64
Diabetes	4 (11%)	2 (20%)	11 (13%)	0.91
Deceased donor	24 (62%)	7 (78%)	43 (52%)	0.57
HLA mm (mean ± SE)	3.3 ± 0.3	3.7 ± 0.7	3.6 ± 0.2	0.54
PRA ≥ 20	8 (22%)	1 (13%)	11 (13%)	0.77
Induction therapy	34 (85%)	10 (100%)	62 (76%)	0.24
C4d positive	2 (5%)	1 (10%)	1 (1%)	<0.0001
Borderline or suspicious for acute cellular rejection	14 (33%)	6 (60%)	2 (2%)	<0.0001
Time to biopsy (median days; interquartile range)	1105; 377–2875	489; 231–1692	376; 362–425	<0.0001
Biopsy >12 months	34 (85%)	6 (60%)	60 (73%)	<0.0001
Death-censored graft loss	14 (35%)	3 (30%)	0	N/A
Time to death-censored graft loss	2935 ± 346	1708 ± 747	N.A.	0.015
Time from biopsy to graft loss	452 ± 189	412 ± 408	N.A.	0.78
Death	6 (15%)	1 (10%)	3 (4%)	N/A
Time to death	1813 ± 385	1324 ± 944	1549 ± 408	0.87

Supplementary Table 4. Demographic and clinical characteristics of the biopsies for clinical indications according to histologic analysis in GSE9492.

Characteristic	STA	IFTA
No. biopsies	1	3
No. patients	1	3
Recipient age (years)	Lost	44.33 ± 11.37

Supplementary Table 5. Demographic and clinical characteristics of the biopsies for clinical indications according to histologic analysis in GSE213714.

Patient demographics	All patients	Failed grafts	Non-failed grafts	*P*
n	*n* = 105	*n* = 30	*n* = 75	
Time of follow-up after biopsy (d)	774	404	922	3×10^{6}
Time of biopsy after transplant (d)	1734	1,853	1,688	0.64
Recipient sex, M/F	65/40	20/10	45/30	0.82
Race				
White	64 (61%)	18 (60%)	46 (61%)	0.99
Black	10 (10%)	4 (13%)	6 (8%)	0.7
Other	15 (14%)	4 (13%)	11 (15%)	0.98
Unknown	16 (15%)	4 (13%)	12 (16%)	0.98
Primary disease				
Diabetic nephropathy	13 (12%)	4 (13%)	9 (12%)	0.98
Hypertension/large-vessel disease	6 (6%)	1 (3%)	5 (7%)	0.8
Glomerulonephritis/vasculitis	51 (49%)	20 (67%)	31 (41%)	0.06
Interstitial nephritis/pyelonephritis	8 (8%)	1 (3%)	7 (9%)	0.58
Polycystic kidney disease	14 (13%)	1 (3%)	13 (17%)	0.16
Others	7 (7%)	2 (7%)	5 (7%)	1
Unknown etiology	6 (6%)	1 (3%)	5 (7%)	0.8
Previous transplant	9 (9%)	0 (0%)	9 (12%)	0.14
Donor sex, M/F	41/64	9/21	32/43	0.49
Deceased donor transplants	57 (54%)	18 (60%)	39 (52%)	0.76
Indication for biopsy				
Rapid deterioration of graft function	26 (25%)	14 (47%)	12 (16%)	0.004
Slow deterioration of graft function	39 (37%)	7 (23%)	32 (43%)	0.18
Stable impaired graft function	7 (7%)	1 (3%)	6 (8%)	0.69
Investigate proteinuria	15 (14%)	7 (23%)	8 (11%)	0.25
Follow-up from previous biopsy	6 (6%)	1 (3%)	5 (7%)	0.8
Others	6 (6%)	0 (0%)	6 (8%)	0.28
Indication unknown	6 (6%)	0 (0%)	6 (8%)	0.28
Estimated GFR (average ml/min ± SD)	47 ± 18	38 ± 20	50 ± 17	0.002
Proteinuria-positive	57 (54%)	25 (83%)	32 (43%)	<0.001
HLA antibody status PRA-positive	64 (61%)	21 (70%)	43 (57%)	0.49
Polycystic kidney disease	14 (13%)	1 (3%)	13 (17%)	0.16
PRA class I–positive	44 (42%)	12 (40%)	32 (43%)	0.97
PRA class II–positive	48 (46%)	16 (53%)	32 (43%)	0.61
DSA-positive	48 (46%)	18 (60%)	30 (40%)	0.18
DSA class I–positive	18 (17%)	7 (23%)	11 (15%)	0.57
DSA class II–positive	38 (36%)	15 (50%)	25 (33%)	0.28
Maintenance immunosuppressive regimens at biopsy				
MMF, tacrolimus, steroid	37 (35%)	11 (37%)	26 (35%)	0.98
MMF, tacrolimus	1 (1%)	1 (3%)	0 (0%)	0.28
MMF, cyclosporine, steroid	26 (25%)	7 (23%)	19 (25%)	0.98
MMF, steroids	3 (3%)	0 (0%)	3 (4%)	0.54
Azathioprine, cyclosporine, steroids	20 (19%)	5 (17%)	15 (20%)	0.93
Others	18 (17%)	6 (20%)	12 (16%)	0.89

[1]Some data were missing

Please browse Full Text version to see the data of Supplementary Table 6:

Supplementary Table 6. Genetic and biological functions of the key genes.
