# Supplementary material for: Profiling of mRNA of interstitial fibrosis and tubular atrophy with subclinical inflammation in recipients after kidney transplantation
Source: Aging (Albany NY). 2019 Jul 25;11(14):5215–31. doi: 10.18632/aging.102115 (PMC6682514; doi:10.18632/aging.102115)
Supplement: Supplementary Table 6 [file aging-11-102115-s001.docx]

**Supplementary Table 6. Genetic and biological functions of the key genes.**

| **Gene** | **Aliases** | **Gene function** |
| --- | --- | --- |
| TAGAP | T-cell activation Rho GTPase-activating protein | May function as a GTPase-activating protein and may play important roles during T-cell activation. |
| IL7R | nterleukin-7 receptor subunit alpha | Receptor for interleukin-7. Also acts as a receptor for thymic stromal lymphopoietin (TSLP). Belongs to the type I cytokine receptor family. Type 4 subfamily. |
| CASP1 | Caspase-1 | Thiol protease that cleaves IL-1 beta between an Asp and an Ala, releasing the mature cytokine which is involved in a variety of inflammatory processes. Important for defense against pathogens. Cleaves and activates sterol regulatory element binding proteins (SREBPs). Can also promote apoptosis. Upon inflammasome activation, during DNA virus infection but not RNA virus challenge, controls antiviral immunity through the cleavage of MB21D1/cGAS, rendering it inactive. Belongs to the peptidase C14A family. |
| CLEC2B | C-type lectin domain family 2 member B | This gene encodes a member of the C-type lectin/C-type lectin-like domain (CTL/CTLD) superfamily. Members of this family share a common protein fold and have diverse functions, such as cell adhesion, cell-cell signalling, glycoprotein turnover, and roles in inflammation and immune response. The encoded type 2 transmembrane protein may function as a cell activation antigen. An alternative splice variant has been described but its full-length sequence has not been determined. This gene is closely linked to other CTL/CTLD superfamily members on chromosome 12p13 in the natural killer gene complex region. |
| LEF1 | Lymphoid enhancer-binding factor 1 | Participates in the Wnt signaling pathway. Activates transcription of target genes in the presence of CTNNB1 and EP300. May play a role in hair cell differentiation and follicle morphogenesis. TLE1, TLE2, TLE3 and TLE4 repress transactivation mediated by LEF1 and CTNNB1. Regulates T-cell receptor alpha enhancer function. Binds DNA in a sequence-specific manner. PIAG antagonizes both Wnt-dependent and Wnt-independent activation by LEF1 (By similarity). Isoform 3 lacks the CTNNB1 interaction domain and may be an antagonist for Wnt signaling. |
| CRTAM | Cytotoxic and regulatory T-cell molecule | Interaction with CADM1 promotes natural killer (NK) cell cytotoxicity and interferon-gamma (IFN-gamma) secretion by CD8+ cells in vitro as well as NK cell-mediated rejection of tumors expressing CADM3 in vivo. Belongs to the nectin family. |
| ITK | Tyrosine-protein kinase ITK/TSK | Tyrosine kinase that plays an essential role in regulation of the adaptive immune response. Regulates the development, function and differentiation of conventional T-cells and nonconventional NKT-cells. When antigen presenting cells (APC) activate T-cell receptor (TCR), a series of phosphorylation lead to the recruitment of ITK to the cell membrane, in the vicinity of the stimulated TCR receptor, where it is phosphorylated by LCK. Phosphorylation leads to ITK autophosphorylation and full activation. |
| CCL5 | C-C motif chemokine 5 | Chemoattractant for blood monocytes, memory T-helper cells and eosinophils. Causes the release of histamine from basophils and activates eosinophils. May activate several chemokine receptors including CCR1, CCR3, CCR4 and CCR5. One of the major HIV-suppressive factors produced by CD8+ T-cells. Recombinant RANTES protein induces a dose-dependent inhibition of different strains of HIV-1, HIV-2, and simian immunodeficiency virus (SIV). The processed form RANTES acts as a natural chemotaxis inhibitor and is a more potent inhibitor of HIV-1- infection. |
| PTPRC | Receptor-type tyrosine-protein phosphatase C | Protein tyrosine-protein phosphatase required for T-cell activation through the antigen receptor. Acts as a positive regulator of T-cell coactivation upon binding to DPP4. The first PTPase domain has enzymatic activity, while the second one seems to affect the substrate specificity of the first one. Upon T-cell activation, recruits and dephosphorylates SKAP1 and FYN. Dephosphorylates LYN, and thereby modulates LYN activity (By similarity). Belongs to the protein-tyrosine phosphatase family. Receptor class 1/6 subfamily. |
| TNFSF8 | Tumor necrosis factor ligand superfamily member 8 | Cytokine that binds to TNFRSF8/CD30. Induces proliferation of T-cells. |
| CD52 | CAMPATH-1 antigen | May play a role in carrying and orienting carbohydrate, as well as having a more specific role. CD molecules. |
| FYB | FYN-binding protein 1 | Acts as an adapter protein of the FYN and LCP2 signaling cascades in T-cells. Modulates the expression of interleukin-2 (IL-2). Involved in platelet activation. Prevents the degradation of SKAP1 and SKAP2. May play a role in linking T-cell signaling to remodeling of the actin cytoskeleton. |
| CCR2 | C-C chemokine receptor type 2 | Receptor for the CCL2, CCL7 and CCL13 chemokines. Receptor for the beta-defensin DEFB106A/DEFB106B. Transduces a signal by increasing intracellular calcium ion levels (By similarity). Upon CCL2 ligation, mediates chemotaxis and migration induction through the activation of the PI3K cascade, the small G protein Rac and lamellipodium protrusion (Probable). |
| SH2D1A | SH2 domain-containing protein 1A | Cytoplasmic adapter regulating receptors of the signaling lymphocytic activation molecule (SLAM) family such as SLAMF1, CD244, LY9, CD84, SLAMF6 and SLAMF7. In SLAM signaling seems to cooperate with SH2D1B/EAT-2. Initially it has been proposed that association with SLAMF1 prevents SLAMF1 binding to inhibitory effectors including INPP5D/SHIP1 and PTPN11/SHP-2. However, by simultaneous interactions, recruits FYN which subsequently phosphorylates and activates SLAMF1. Positively regulates CD244/2B4- and CD84- mediated natural killer (NK) cell functions. |
| SAMHD1 | Deoxynucleoside triphosphate triphosphohydrolase SAMHD1 | Host restriction nuclease involved in defense response to virus. Has dNTPase activity and reduces cellular dNTP levels to levels too low for retroviral reverse transcription to occur. Blocks early-stage virus replication in dendritic and other myeloid cells. Likewise, suppresses LINE-1 retrotransposon activity. May play a role in mediating proinflammatory responses to TNF-alpha signaling. Has ribonuclease activity, acting on single-stranded RNA. This activity is essential for H1V-1 restriction. Sterile alpha motif domain containing. |
| NELL2 | Protein kinase C-binding protein NELL2 | Required for neuron survival through the modulation of MAPK pathways (By similarity). Involved in the regulation of hypothalamic GNRH secretion and the control of puberty (By similarity). |
| CD84 | SLAM family member 5 | Self-ligand receptor of the signaling lymphocytic activation molecule (SLAM) family. SLAM receptors triggered by homo- or heterotypic cell-cell interactions are modulating the activation and differentiation of a wide variety of immune cells and thus are involved in the regulation and interconnection of both innate and adaptive immune response. Activities are controlled by presence or absence of small cytoplasmic adapter proteins, SH2D1A/SAP and/or SH2D1B/EAT-2. Can mediate natural killer (NK) cell cytotoxicity dependent on SH2D1A and SH2D1B (By similarity). |
| CYTIP | Cytohesin-interacting protein | By its binding to cytohesin-1 (CYTH1), it modifies activation of ARFs by CYTH1 and its precise function may be to sequester CYTH1 in the cytoplasm. PDZ domain containing. |
| ARL4C | ADP-ribosylation factor-like protein 4C | Small GTP-binding protein which cycles between an inactive GDP-bound and an active GTP-bound form, and the rate of cycling is regulated by guanine nucleotide exchange factors (GEF) and GTPase-activating proteins (GAP). GTP-binding protein that does not act as an allosteric activator of the cholera toxin catalytic subunit. May be involved in transport between a perinuclear compartment and the plasma membrane, apparently linked to the ABCA1-mediated cholesterol secretion pathway. |
| RGS18 | Regulator of G-protein signaling 18 | Inhibits signal transduction by increasing the GTPase activity of G protein alpha subunits thereby driving them into their inactive GDP-bound form. Binds to G(i) alpha-1, G(i) alpha- 2, G(i) alpha-3 and G(q) alpha. Regulators of G-protein signaling. |
| LILRB1 | Leukocyte immunoglobulin-like receptor subfamily B member 1 | Receptor for class I MHC antigens. Recognizes a broad spectrum of HLA-A, HLA-B, HLA-C and HLA-G alleles. Receptor for H301/UL18, a human cytomegalovirus class I MHC homolog. Ligand binding results in inhibitory signals and down-regulation of the immune response. Engagement of LILRB1 present on natural killer cells or T-cells by class I MHC molecules protects the target cells from lysis. Interaction with HLA-B or HLA-E leads to inhibition of the signal triggered by FCER1A and inhibits serotonin release. |
| TRG-AS1 | T Cell Receptor Gamma Locus Antisense RNA 1 | An RNA Gene, and affiliated with the non-coding RNA class. |
| B2M | Beta-2-microglobulin | Component of the class I major histocompatibility complex (MHC). Involved in the presentation of peptide antigens to the immune system. Exogenously applied M.tuberculosis EsxA or EsxA-EsxB (or EsxA expressed in host) binds B2M and decreases its export to the cell surface (total protein levels do not change), probably leading to defects in class I antigen presentation. Belongs to the beta-2-microglobulin family. |
| GZMA | Granzyme A | Abundant protease in the cytosolic granules of cytotoxic T-cells and NK-cells which activates caspase-independent cell death with morphological features of apoptosis when delivered into the target cell through the immunological synapse. It cleaves after Lys or Arg. Cleaves APEX1 after 'Lys-31' and destroys its oxidative repair activity. Cleaves the nucleosome assembly protein SET after 'Lys-189', which disrupts its nucleosome assembly activity and allows the SET complex to translocate into the nucleus to nick and degrade the DNA. Belongs to the peptidase S1 family. |
| CD2 | T-cell surface antigen CD2 | CD2 interacts with lymphocyte function-associated antigen (LFA-3) and CD48/BCM1 to mediate adhesion between T-cells and other cell types. CD2 is implicated in the triggering of T- cells, the cytoplasmic domain is implicated in the signaling function. C2-set domain containing. |
| LOC105369277 | Transmembrane Protein C16orf54 | A Protein Coding gene. |
| IRF8 | Interferon regulatory factor 8 | Plays a role as a transcriptional activator or repressor. Specifically binds to the upstream regulatory region of type I IFN and IFN-inducible MHC class I genes (the interferon consensus sequence (ICS)). Plays a negative regulatory role in cells of the immune system. Involved in CD8(+) dendritic cell differentiation by forming a complex with the BATF-JUNB heterodimer in immune cells, leading to recognition of AICE sequence (5'-TGAnTCA/GAAA-3'), an immune-specific regulatory element, followed by cooperative binding of BATF and IRF8 and activation of genes (By similarity). Positively regulates macroautophagy in dendritic cells. |
| GZMK | Granzyme K | Belongs to the peptidase S1 family. Granzyme subfamily. |
| RGS1 | Regulator of G-protein signaling 1 | Regulates G protein-coupled receptor signaling cascades, including signaling downstream of the N-formylpeptide chemoattractant receptors and leukotriene receptors. Inhibits B cell chemotaxis toward CXCL12 (By similarity). Inhibits signal transduction by increasing the GTPase activity of G protein alpha subunits thereby driving them into their inactive GDP-bound form. |
| KLRC4 | NKG2-F type II integral membrane protein | May play a role as a receptor for the recognition of MHC class I HLA-E molecules by NK cells. Killer cell lectin like receptors. |
| KLRB1 | Killer cell lectin-like receptor subfamily B member 1 | Plays an inhibitory role on natural killer (NK) cells cytotoxicity. Activation results in specific acid sphingomyelinase/SMPD1 stimulation with subsequent marked elevation of intracellular ceramide. Activation also leads to AKT1/PKB and RPS6KA1/RSK1 kinases stimulation as well as markedly enhanced T-cell proliferation induced by anti-CD3. Acts as a lectin that binds to the terminal carbohydrate Gal-alpha(1,3)Gal epitope as well as to the N-acetyllactosamine epitope. Binds also to CLEC2D/LLT1 as a ligand and inhibits NK cell-mediated cytotoxicity as well as interferon-gamma secretion in target cells. |
| NLRC3 | NLR family CARD domain-containing protein 3 | Negative regulator of the innate immune response. Attenuates signaling pathways activated by Toll-like receptors (TLRs) and the DNA sensor STING/TMEM173 in response to pathogen-associated molecular patterns, such as intracellular poly(dA:dT), but not poly(I:C), or in response to DNA virus infection, including that of Herpes simplex virus 1 (HSV1) (By similarity). May affect TLR4 signaling by acting at the level of TRAF6 ubiquitination, decreasing the activating 'Lys-63'- linked ubiquitination and leaving unchanged the degradative 'Lys- 48'-l-linked ubiquitination (PubMed:22863753). Inhibits the PI3K-AKT-mTOR pathway possibly by directly interacting with the posphatidylinositol 3-kinase regulatory subunit p85 (PIK3R1/PIK3R2) and disrupting the association between PIK3R1/PIK3R2 and the catalytic subunit p110 (PIK3CA/PIK3CB/PIK3CD) and reducing PIK3R1/PIK3R2 activation. Via its regulation of the PI3K-AKT-mTOR pathway, controls cell proliferation, predominantly in intestinal epithelial cells (By similarity). May also affect NOD1- or NOD2-mediated NF-kappa-B activation (PubMed:25277106). Might also affect the inflammatory response by preventing NLRP3 inflammasome formation, CASP1 cleavage and IL1B maturation. |
| CD53 | Leukocyte surface antigen CD53 | Required for efficient formation of myofibers in regenerating muscle at the level of cell fusion. May be involved in growth regulation in hematopoietic cells (By similarity). CD molecules. |
